# Effects of a Si-doped InGaN Underlayer on the Optical Properties of InGaN/GaN Quantum Well Structures with Different Numbers of Quantum Wells

**DOI:** 10.3390/ma11091736

**Published:** 2018-09-15

**Authors:** George Christian, Menno Kappers, Fabien Massabuau, Colin Humphreys, Rachel Oliver, Philip Dawson

**Affiliations:** 1 School of Physics and Astronomy, Photon Science Institute, University of Manchester, Manchester M13 9PL, UK; george.christian@manchester.ac.uk; 2 Department of Materials Science and Metallurgy, University of Cambridge, Cambridge CB3 0FS, UK; mjk30@cam.ac.uk (M.K.); fm350@cam.ac.uk (F.M.); c.humphreys@qmul.ac.uk (C.H.); 3 School of Engineering and Materials Science, Queen Mary University of London, London E1 4NS, UK

**Keywords:** photoluminescence, InGaN, underlayer, quantum well, radiative lifetime

## Abstract

In this paper we report on the optical properties of a series of InGaN polar quantum well structures where the number of wells was 1, 3, 5, 7, 10 and 15 and which were grown with the inclusion of an InGaN Si-doped underlayer. When the number of quantum wells is low then the room temperature internal quantum efficiency can be dominated by thermionic emission from the wells. This can occur because the radiative recombination rate in InGaN polar quantum wells can be low due to the built-in electric field across the quantum well which allows the thermionic emission process to compete effectively at room temperature limiting the internal quantum efficiency. In the structures that we discuss here, the radiative recombination rate is increased due to the effects of the Si-doped underlayer which reduces the electric field across the quantum wells. This results in the effect of thermionic emission being largely eliminated to such an extent that the internal quantum efficiency at room temperature is independent of the number of quantum wells.

## 1. Introduction

There have been several recent investigations [1,2,3,4,5,6,7,8,9,10,11,12,13,14] into the effects of the inclusion of layers grown prior to the first quantum well (QW) in InGaN/GaN light-emitting diodes (LEDs) and in structures for photoluminescence (PL) experiments. These layers, referred to as underlayers (ULs), are often layers of n-doped InGaN with a low In content and their inclusion has been reported to give rise to a variety of improvements including increased PL or electroluminescence (EL) intensity at low temperature [1,2,3,4] and at room temperature [5,6,7] as well as increased room temperature internal quantum efficiency (IQE) [1,2,8,9,11,13]. However, the mechanism(s) responsible for these improvements remain the subject of debate. It has been demonstrated [14,15,16,17] that the spontaneous polarization in group III-nitrides leads to a depletion field that is in the opposite sense to the fields across the QWs caused by the piezoelectric polarization. Also, it has been shown that the Fermi level pinning caused by n-type doping can be used to manipulate the strength of this field close to the surface of a GaN structure [12,13,14,16,17], and that this affects the emission properties of QWs grown in this region through modifications to the quantum confined Stark effect (QCSE) [12,13,14,17]. It has been suggested that the efficiency improvements reported for structures containing ULs are therefore due to the increased electron-hole wave function overlap leading to an increased radiative recombination rate following a decrease in the electric field across the QWs [11,12,13]. Usually this occurs because of the more effective competition with non-radiative processes associated with defects of impurities. However, it should be noted that it has also been proposed that the addition of ULs to a structure leads to a reduced impurity incorporation at interfaces [18] similar to observations first made [19] in GaAs/AlGaAs heterostructures.

In general the benefits on the IQE of QW structures have been discussed in terms of the reduction in the effectiveness of non-radiative pathways associated with defects or impurities rather than any effects associated with the design of the QW structures. It has already been demonstrated that the room temperature IQE of QW structures can be strongly influenced by varying the number of QWs [20,21]. It particular, it has been shown that thermionic emission of carriers from QWs can be the efficiency-limiting process in single GaAs/AlGaAs QW structures [21] and in single InGaN/GaN QWs. This effect can be overcome in multiple quantum well (MQW) structures due to the recapture of the photo-excited carriers by other QWs in the stack [20]. It is the purpose of this paper to determine whether the effects of thermionic emission can be overcome by the inclusion of ULs which lead to a reduced radiative recombination rate to such an extent that the thermally driven escape of carriers can be negated.

## 2. Sample Details and Experimental Techniques

Six InGaN/GaN QW samples containing ULs were grown by Metal Organic Chemical Vapor Deposition (MOCVD) for these investigations using the two-temperature (2T) growth method [22]. The samples were grown on top of 5 µm thick GaN pseudo-substrates on sapphire. For all of the samples, a 2 µm thick GaN:Si template with nominal doping density 5 × 10^18^ cm^−3^ was grown followed by a 23 nm In_0.05_Ga_0.95_N:Si UL with the same nominal doping density. On top of the UL, a 3 nm layer of undoped GaN was grown followed by the QW(s) and barriers(s). The number of QW-barrier periods was varied between 1 and 15. The QW widths, In fractions and barrier thicknesses were determined by X-ray diffraction (XRD) measurements using a Philips X’ Pert system. However the 2T growth method leads to gross well width fluctuations [22] and thus we adopted a variant to the original XRD methodology used by Vickers et al. [23]. Using an ω-2θ scan of the symmetric 002 reflection, XRD can accurately determine the thickness of a QW + barrier repeat, and the average In fraction reported over the entire QW + barrier repeat. Unique determination of the QW width, In fraction and barrier thickness relies on the presence of a missing satellite peak in such a scan [23]. However, there is no such missing peak when QWs exhibit substantial variations in thickness [24]. To obtain the QW width and composition, two additional 5 QW samples were grown using the same growth conditions except that the GaN barrier width was varied. From the determination of the QW + barrier thickness of these samples, we were then in position to extrapolate an “effective” QW width (which is in essence the average width of the QW) and its “effective” QW composition. The effective QW width thus obtained was 2.3 ± 0.1 nm and the In fraction for each sample is listed in Table 1. Atomic force microscopy (AFM) measurements showed no systematic variation in the density of V-pits on the surfaces of the samples.

The optical properties were studied using a combination of continuous wave PL spectroscopy and PL decay time measurements. The samples were mounted on the cold finger of a temperature-controlled closed-cycle Advance Research Systems helium cryostat (Macungie, PA, USA). For the time decay measurements, the samples were excited using the frequency tripled output (wavelength 266 nm) of a 100 fs mode locked Spectra Physics Ti:sapphire laser (Santa Clara, CA, USA) such that the energy per pulse was ~1 µJ cm^−2^ at the sample surface. The continuous wave PL spectroscopy was performed by modulating the light from either a He/Cd laser or a diode laser with a chopper and by dispersing the PL through a 0.75 m Spex single grating spectrometer which was then detected by an RCA GaAs photomultiplier tube; the signal from the photomultiplier was then processed by a Stanford Research Systems lock-in detector (Sunnyvale, CA, USA). For the PL time-decay measurements, a Hamamatsu micro-channel plate detector (Hamamatsu Photonics, Hamamatsu, Japan) was used to detect the PL via the spectrometer and the signal was processed using the technique of time-correlated single photon counting.

## 3. Results

### 3.1. Simulation of Conduction and Valence Band Profiles

**Figure 1:**
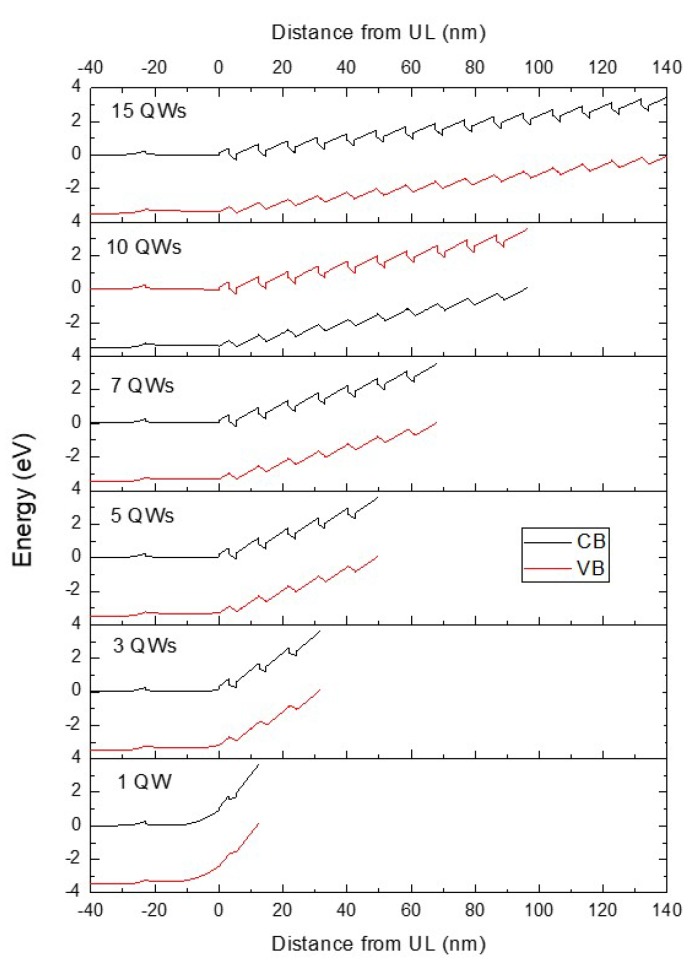
Results of calculations of conduction band (CB) and valence band (VB) edges for the samples with different numbers of QWs.

The simulated conduction band (CB) and valence band (VB) profiles for the sample series are shown in Figure 1 and were calculated using the commercial package Nextnano [25],. We assume that the In fraction is the same for all the QWs in any one structure. The position axis is defined with the positive (growth) direction towards the sample surface where zero is set at the interface between the UL and the bottom QW barrier. The energy axis is defined with the Fermi level at zero. As was reported by Davies et al. [11,12,13], the Fermi level is pinned at the CB edge in the region of the UL and at the VB edge at the sample surface where the material is undoped [16,17]. The strength of the resulting surface depletion field, corresponding to the overall gradient between these points, decreases with increasing number of QWs. The electric fields across each QW for each of the samples were calculated from the simulated band edge profiles plotted in Figure 1.

**Figure 2:**
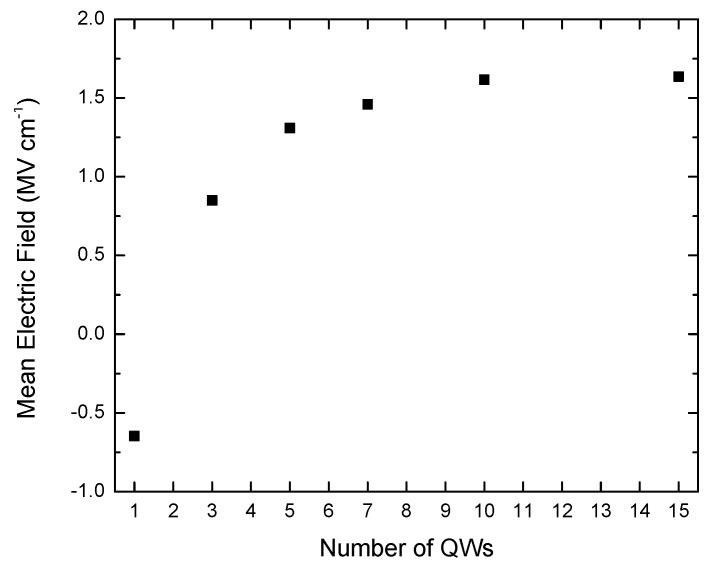
Calculated mean electric field across the QWs as a function of the number of QWs in the different samples.

Figure 2 shows that the electric field for the 1 QW sample is negative to indicate that it is in the opposite direction to that in the rest of the samples. The mean electric field across the QWs increases with increasing number of QWs (Figure 2), varying from −0.608 MV cm^−1^ for the 1 QW sample to 1.64 MV cm^−1^ for the 15 QW sample. For all of the QWs in all of the samples with more than 1 QW, apart from the first QW in the 10 and 15 QW samples, the electric field strength varies by less than 1% between the QWs for a particular sample. For the 10 QW and 15 QW samples, the electric field across the first QW in the stack is reduced relative to the second QW by 0.0468 and 0.108 MV cm^−1^, or 2.9 and 6.6%, respectively. This is due to an additional sheet charge that occurs at the In_0.05_Ga_0.95_N/GaN interface between the UL and the first QW barrier due to the large polarization discontinuity between the two layers, and which is of opposite sense to the sheet charge formed at the GaN/InGaN interface at the bottom of the first QW in the stack [11,12,13]. 

The In content of the 1 QW sample is significantly smaller compared with the rest of the samples in the series; as shown in Table 1, the measured In fractions of the QWs in the 1 QW and 3 QW samples are (8 ± 1)% and (11 ± 1)%, respectively. The reduced In fraction in the 1 QW sample means that there is a reduced contribution from the strain-induced piezoelectric field. This would be in addition to the net field reduction effect provided by the surface depletion field. Nevertheless, the UL provides a major contribution to the change in net electric field between the 1 and 3 QW samples. The calculated mean electric field for all the samples is shown in Figure 2.

### 3.2. Low-Temperature Photoluminescence (PL) Spectroscopy

**Figure 3:**
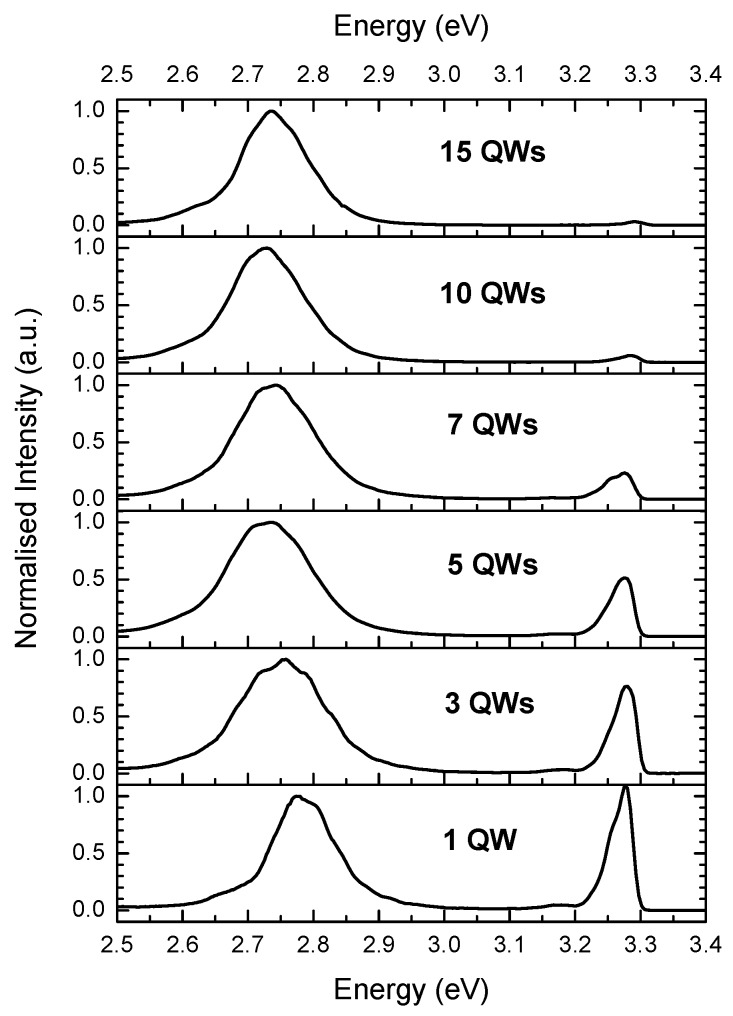
Photoluminescence (PL) spectra measured at 10K for the different samples with the number of QWs as indicated.

Normalized PL spectra obtained at 10 K using continuous-wave (CW) excitation at 3.185 eV, with a power density at the sample of 15 W cm^−2^, are shown for each sample in Figure 3. Two main peaks are observed for all of the samples, one with a peak energy in the range at 3.28–3.29 eV and another in the range 2.74–2.79 eV. The lower energy peak is attributed to radiative recombination in the QW(s). The structure in the QW emission feature is due to the effects of optical interference caused by the finite reflectivity of the GaN/air and GaN/sapphire interfaces. The higher energy peak is due to emission from the In_0.05_Ga_0.95_N:Si UL [11]. The PL intensities in Figure 3 have been normalized to the peak of the QW emission.

The peak PL emission energy from the QW(s) red shifts as the number of QWs is increased from 3 to 10. This may reflect the possibility that the change in the predicted magnitude of the electric field strength across the QWs decreases with increasing number of QW periods. We cannot make a firm conclusion on this aspect of the spectra due any changes that may occur in the effects of carrier localization that play a major role in the recombination energy of light emitted from InGaN/GaN QWs. Also there is a significant uncertainty of ±0.01 in the measured values of the In fractions. This latter point is especially valid for the red shift between the peak emission energies of the 1 QW and 3 QW samples where there is a distinct change in the measured In fractions in the two samples. 

The peak emission energy for the 15 QW sample is (10 ± 2) meV higher than that for the 10 QW sample. From the simulated band profiles, which were calculated for fully strained QWs, a lower energy emission peak for the 15 QW sample relative to the 10 QW sample would be expected due to the slightly higher mean electric field across the QWs. It has been shown that strain relaxation can occur in low In fraction InGaN/GaN MQW structures with around 10 or more QWs [26]. It is, therefore, possible that strain relaxation has occurred in the 15 QW sample, in which case the mean electric field across the QWs would be reduced. This would lead to a reduced QCSE giving greater emission peak energy than for the fully strained case.

### 3.3. Photoluminescence Time-Decay Measurements

**Figure 4:**
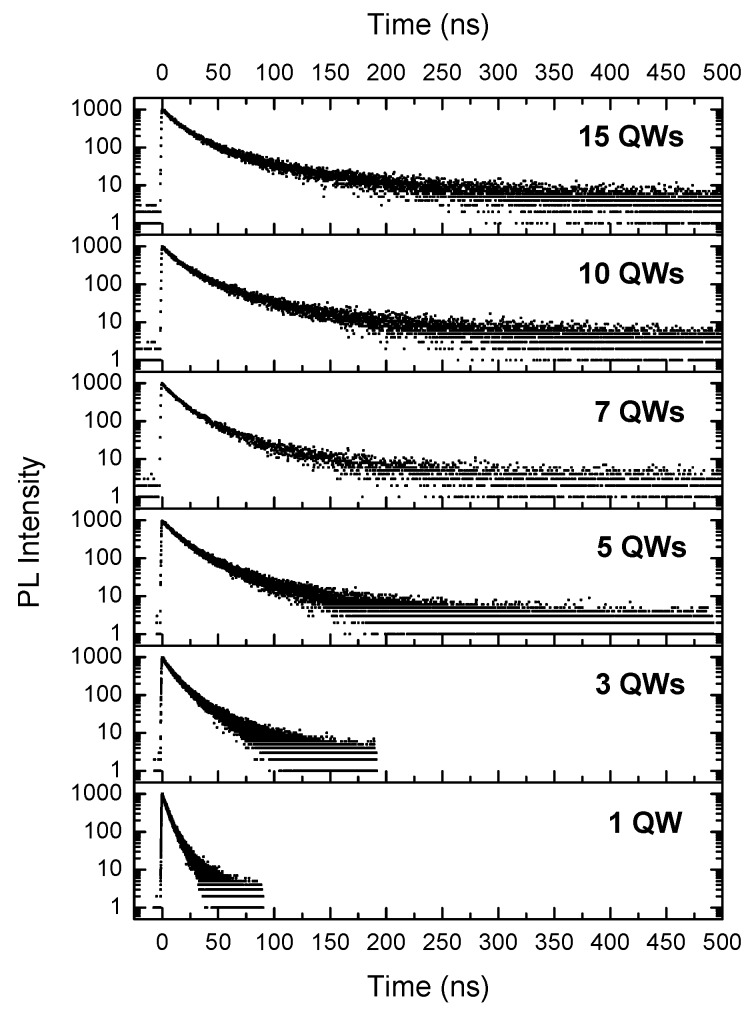
PL time decays when measuring at the peak of the emission at a temperature of 10 K for the different samples with the number of QWs as indicated.

PL time-decay measurements were performed on each of the samples. The PL time-decay curves detected at the peak of the QW emission for each sample are shown in Figure 4. It has been widely reported [11,27,28,29,30,31] that decay curves for polar InGaN/GaN QWs are non-exponential at low temperatures. 

Therefore, the time taken for the PL intensity to reach 1/e of its peak value is used as an arbitrary measure of the decay time and is shown as a function of the number of QWs in Table 2. At 10 K, the value of 1/e is assumed to be governed entirely by radiative recombination [31]. In general, a trend of increasing PL decay time with increasing number of QWs is observed. This agrees with the variation in electric field strength across the QWs determined from the calculated energy band profiles. That is, the mean strength of the electric field across the QWs increases with increasing number of QW periods as the strength of the surface depletion field is reduced. This causes the electron-hole wave function overlap to decrease, resulting in a decreasing radiative recombination rate.

### 3.4. Temperature-Dependent Photoluminescence Spectroscopy

**Figure 5:**
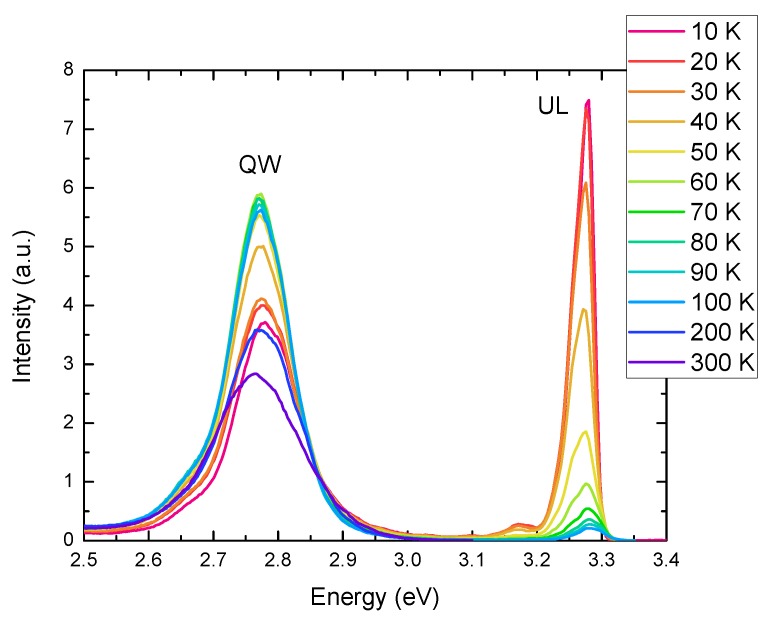
PL spectra for the 1 QW sample as a function of temperature.

Temperature-dependent PL spectroscopy was performed using CW excitation above the band gap of the GaN barriers for each of the samples at an excitation power density of 15 W cm^−2^. A selection of the PL spectra taken over the temperature range 10–300 K is shown for the 1 QW sample in Figure 5.

**Figure 6:**
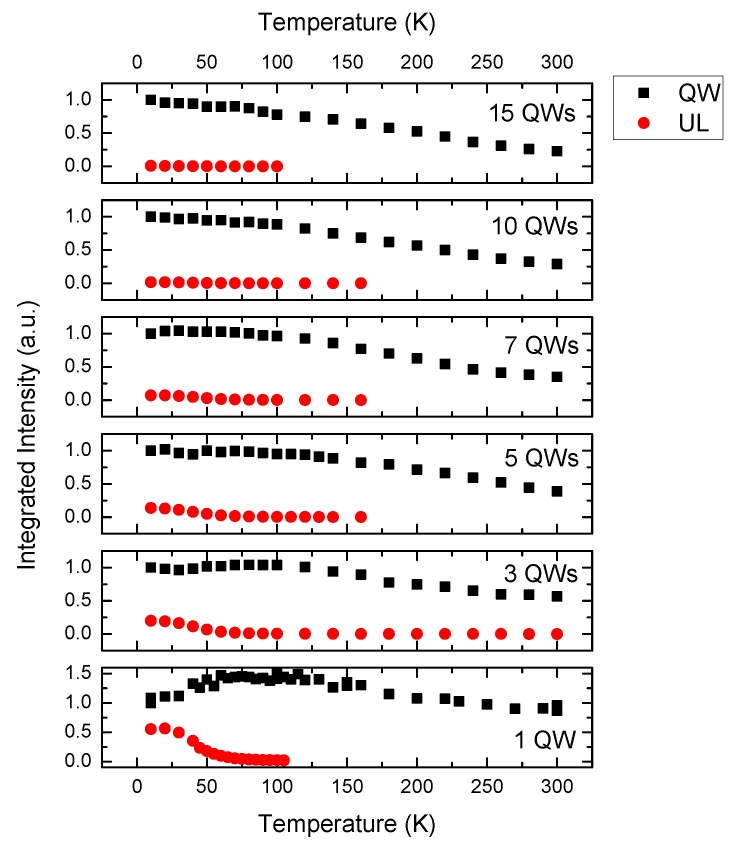
Integrated intensity of the underlayer (UL) emission from the QW and the UL as a function of temperature for all the samples. The emission intensities are normalized to the emission intensity for each sample at 10 K.

This general behavior whereby the UL emission collapses as well as an increase in the QW emission is seen in all samples with increasing temperature. The temperature dependences of the integrated intensities of the QW and UL emission bands for the 1 QW sample are shown in Figure 6. The integrated intensity values are normalized to that of the QW peak at 10 K.

**Figure 7:**
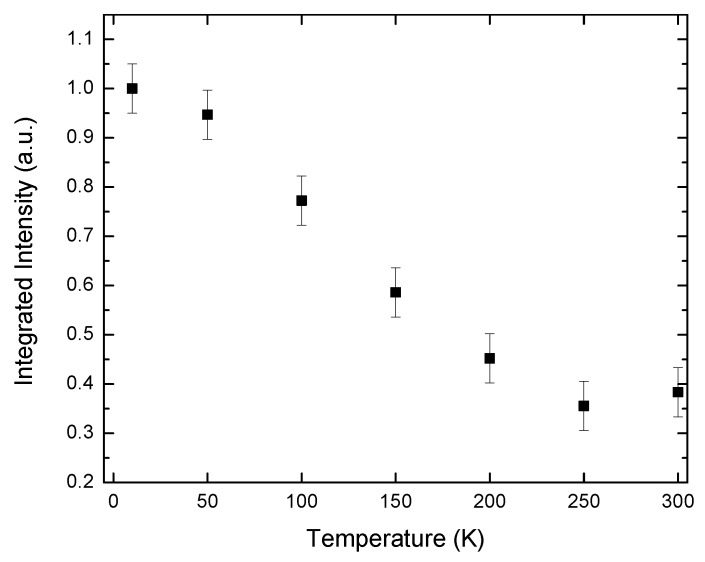
Temperature dependence of the integrated intensity of the QW emission for the 1 QW samples when excited by a diode laser where the excitation photon energy was 3.061 eV.

The UL emission clearly plays a role in the spectra for the 1 QW, 3 QW, 5 QW and 7 QW samples, the intensity of the UL emission is relatively very small in the 10 QW and 15 QW samples. The UL emission is strongest relative to the QW emission in the 1 QW sample. The integrated intensity of the UL emission peak falls rapidly from 0.55 a.u. to zero as the temperature is increased from 10 to 100 K. However, the integrated intensity of the QW emission peak increases by 0.45 a.u. over the same temperature range. The integrated intensity of the QW emission reaches a maximum around the temperature where the UL emission has gone to zero, and then it decreases monotonically up to 300 K to a value that is ~50% less than its value at 100 K. We attribute the intensity increase and decrease for the QW and the UL emission channels, respectively, to a process whereby carriers that are captured or excited in the UL are thermally transferred to the QW. This argument applies to the 1 QW, 3 QW, 5 QW and 7 QW samples. It is not a problem in the 10 QW and 15 QW samples as most of the excitation radiation is absorbed by the QWs before it reaches the UL. In support of this assignment this increase in the QW emission as a function of temperature is not observed when the structure is excited with light from a diode laser whose photon energy lies below the UL emission energy but above the QW emission energy. This is shown for the 1 QW sample in Figure 7.

While the lifetime data shown in Figure 4 demonstrate that the radiative rate is modified by the inclusion of the UL, the question remains how this influences the IQE at room temperature. As discussed in the introduction, it has been shown that the inclusion of MQWs can overcome the effects of thermionic emission. However, if the radiative rate is itself increased to the large extent shown in Figure 4 it may not be necessary to include many QWs in the “active” region as the radiative process can compete effectively with the thermionic emission process. This may be beneficial in structures where the uneven distribution of carriers across the QW region is a key issue [32,33]. In PL experiments it has become the widespread practice [34,35,36] to determine the room temperature IQE by assuming the IQE at low temperature (e.g., 10 K) is 1.0 and then referencing the PL integrated intensity at high temperature to the low temperature value; thus determining the high temperature IQE. Clearly this is not appropriate for the samples studied here as the QW-integrated PL intensity increases as carriers are transferred from the UL. So as some measure of the high temperature IQE we assume that the IQE at 100 K (where the influence of the carriers excited in the UL is negligible) is ~1.0. Using this procedure we obtain the following measures shown in Table 3 of IQE at 300 K for the samples with the different numbers of QWs.

## 4. Discussion and Conclusions

The data shown in Table 3 clearly suggest that the effects of thermionic emission at room temperature have been negated by the presence of the UL in the samples, since the IQEs of the 1 QW and 3 QW structures are in fact higher than those of the other samples in the series, whereas if thermionic emission were dominating, we would expect them to be lower. We propose that this occurs because of the reduction in the QCSE and the subsequent increase in the radiative rate is sufficient to overcome the effects of thermionic emission. As for the rest of the sample series, the change in radiative rate is relatively small and indeed the IQE decreases somewhat with increasing number of wells. As the measurements were performed with a constant excitation power density, the carrier density per QW decreases as the number of wells increases in the different samples, thus possibly allowing non-radiative recombination at defects or impurities to become more effective with decreasing carrier density [34,37,38,39,40] in the different samples.

## Figures and Tables

**Table 1:** List of number of quantum wells (QWs) in each sample with effective In fraction in the QWs.

Number of QWs	QW In Fraction (±0.01)
1	0.08
3	0.11
5	0.12
7	0.12
10	0.12
15	0.12

**Table 2:** 1/e lifetimes extracted from the data in Figure 4 for the different number of QW samples.

Number of QWs	1/e Time (ns)
1	5
3	10
5	15
7	15
10	16
15	17

**Table 3:** Extracted values of internal quantum efficiency (IQE) at 300K for the samples with different numbers of QWs.

Number of QWs	IQE (300K)
1	0.56
3	0.55
5	0.4
7	0.36
10	0.33
15	0.29
